# Risk of Herpes Zoster Among Psoriasis Patients Taking Biologics: A Network Meta-Analysis of Cohort Studies

**DOI:** 10.3389/fmed.2021.665559

**Published:** 2021-06-04

**Authors:** Zhenwei Tang, Minxue Shen, Xiang Chen

**Affiliations:** ^1^Department of Dermatology, Xiangya Hospital, Central South University, Changsha, China; ^2^Hunan Engineering Research Center of Skin Health and Disease, Hunan Key Laboratory of Skin Cancer and Psoriasis (Xiangya Hospital), Changsha, China; ^3^Department of Social Medicine and Health Management, Xiangya School of Public Health, Central South University, Changsha, China

**Keywords:** psoriasis, biologics, herpes zoster, infection, cohort studies

## Abstract

**Background:** Herpes zoster (HZ) has raised public concern. An increasing incidence of HZ can be seen in the immunocompromised population, such as the psoriasis patients taking biologics. Real-world evidences are still needed to investigate the risks of HZ among patients receiving different biologics treatments. This study aims to summarize the findings from cohort studies.

**Methods:** Herein, we performed a meta-analysis of cohort studies. We included studies referred to seven biologics (adalimumab, alefacept, efalizumab, etanercept, infliximab, rituximab, and ustekinumab) as well as methotrexate for psoriasis. We estimated summary relative risks (RRs) for HZ using pairwise and network meta-analysis.

**Results:** Overall, five studies were included for analysis. A total of 32827.6 patient-years were observed. The result of the meta-analysis showed that the pooled HZ incidence rate of adalimumab, which accounts for the most patient-years in our analysis, is 2.6 per 1,000 patient-years. Our analysis based on several cohorts showed an insignificant difference among the patients receiving adalimumab, alefacept, efalizumab, etanercept, infliximab, rituximab, ustekinumab, and methotrexate.

**Conclusions:** Based on this analysis, the type of mono-biologic treatment contributes little to the risk of HZ among psoriasis patients. Of note, the negative findings of our study do not mean the unnecessity of vaccination. More efforts must be taken to further determine HZ risk of different therapeutic strategies.

## Introduction

Psoriasis is a chronic autoimmune disease, manifesting with featured skin lesions and systematic disorders. Besides, the substantial negative effects on patient quality of life increase the burden of psoriasis (1). Recently, the application of biologics which function *via* immunomodulation has brought surprising effects for psoriasis management, compared to conventional treatment (2). However, some potential side effects of biologics have been noticed such as the increasing risks of infection (3). Evaluation of the potential risks of psoriasis patients receiving biologics to get infected by various pathogens will help and guide a better management of psoriasis patients.

Herpes zoster (HZ) has raised public concern due to potential postherpetic neuralgia after its occurrence (4, 5). An increasing incidence of HZ can be seen in the immunocompromised population, such as the psoriasis patients taking biologics. Although more and more clinical trials are emerging, they often suffer from short-term observation or selection bias. Real-world evidences are still needed to investigate the risks of HZ among patients receiving different biologics treatment. To provide a comprehensive comparison of the HZ risk among psoriasis patients taking different biologics, we performed a meta-analysis of cohort studies.

## Methods

### Search Strategy and Selection Criteria

This study has been registered on PROSPERO (CRD42020214956). We searched PubMed, MEDLINE, Embase, and Cochrane library from database inception to October 19, 2020, using the following search terms: “psoriasis,” “biologics,” “infection,” “hepes zoster,” “biological agents,” “Infliximab,” “Adalimumab,” “Ixekizumab,” “Risankizumab,” “Brodalumab,” “Secukinumab,” “mirikizumab,” “Ustekinumab,” “Guselkumab,” and “Tocilizumab.” An example for search in PubMed: (“infect”[All Fields] OR “infectability”[All Fields] OR “infectable”[All Fields] OR “infectant”[All Fields] OR “infectants”[All Fields] OR “infected”[All Fields] OR “infecteds”[All Fields] OR “infectibility”[All Fields] OR “infectible”[All Fields] OR “infecting”[All Fields] OR “infection s”[All Fields] OR “infections”[MeSH Terms] OR “infections”[All Fields] OR “infection”[All Fields] OR “infective”[All Fields] OR “infectiveness”[All Fields] OR “infectives”[All Fields] OR “infectivities”[All Fields] OR “infects”[All Fields] OR “pathogenicity”[MeSH Subheading] OR “pathogenicity”[All Fields] OR “infectivity”[All Fields] OR (“virology”[MeSH Subheading] OR “virology”[All Fields] OR “viruses”[All Fields] OR “viruses”[MeSH Terms] OR “virus s”[All Fields] OR “viruse”[All Fields] OR “virus”[All Fields]) OR (“tuberculosi”[All Fields] OR “tuberculosis”[MeSH Terms] OR “tuberculosis”[All Fields] OR “tuberculoses”[All Fields] OR “tuberculosis s”[All Fields])) AND “psoriasis”[Title/Abstract] AND (“biological agents”[Title/Abstract] OR “biologics”[Title/Abstract] OR “Infliximab”[Title/Abstract] OR “Adalimumab”[Title/Abstract] OR “Ixekizumab”[Title/Abstract] OR “Risankizumab”[Title/Abstract] OR “Brodalumab”[Title/Abstract] OR “mirikizumab”[Title/Abstract] OR “Secukinumab”[Title/Abstract] OR “Ustekinumab”[Title/ Abstract] OR “Guselkumab”[Title/Abstract] OR “Tocilizumab”[Title/Abstract]).

Two investigators (Z.T. and M.S.) independently searched the databases. We identified cohort studies containing monotherapy of biologics for patients who fulfilled the diagnostic criteria for psoriasis. We included published cohort studies with no language restrictions to limit publication bias. When duplicate publications were identified, we included only the report with the most comprehensive data. We excluded studies if they used a regimen other than the strategies as aforementioned or contained no clear information about events of HZ. Studies that met inclusion criteria were retrieved for full-text evaluation. Any discrepancies and disagreements were resolved with the consensus of all investigators.

### Data Extraction and Risk of Bias Assessment

Two reviewers (Z.T. and M.S.) independently extracted information from each selected study. Any disagreements were resolved by a third investigator (X.C.). We extracted incidence rates (RRs) for HZ and total patient-years of patients receiving a certain type of biologic. The potential publication bias (small study effects) was estimated using visual inspection of funnel plots and corresponding Egger's regression test. *P*-values <0.10 correspond to statistically significant publication bias.

### Statistical Analysis

A network meta-analysis was performed for each outcome that was computed in a Bayesian framework by R version 3.4.2. Both time-to-event estimates and dichotomous were calculated respectively by fixed-effects and random-effects models, and we used posterior mean of residual deviance and deviance information criteria to access the fit of each model. Models were computed with Markov chain Monte Carlo simulations. For each outcome, three independent Markov chains with over-dispersed initial values from −2.50 to 2.50 were run with 100,000 inference iterations and a thinning interval of 10 per chain after a burn-in phase of 20,000 iterations to estimate the posterior distributions of parameters. Convergence of iterations was assessed graphically according to Gelman and Rubin. Inconsistency was evaluated by comparing direct and indirect evidence on a specific node (the split node) from the entire network, and *P*-values < 0.1 were considered to be significant in inconsistency evaluation. The heterogeneity between trials as measured by a random-effects model was evaluated by the estimate of the corresponding standard deviation.

## Results

**Figure 1:**
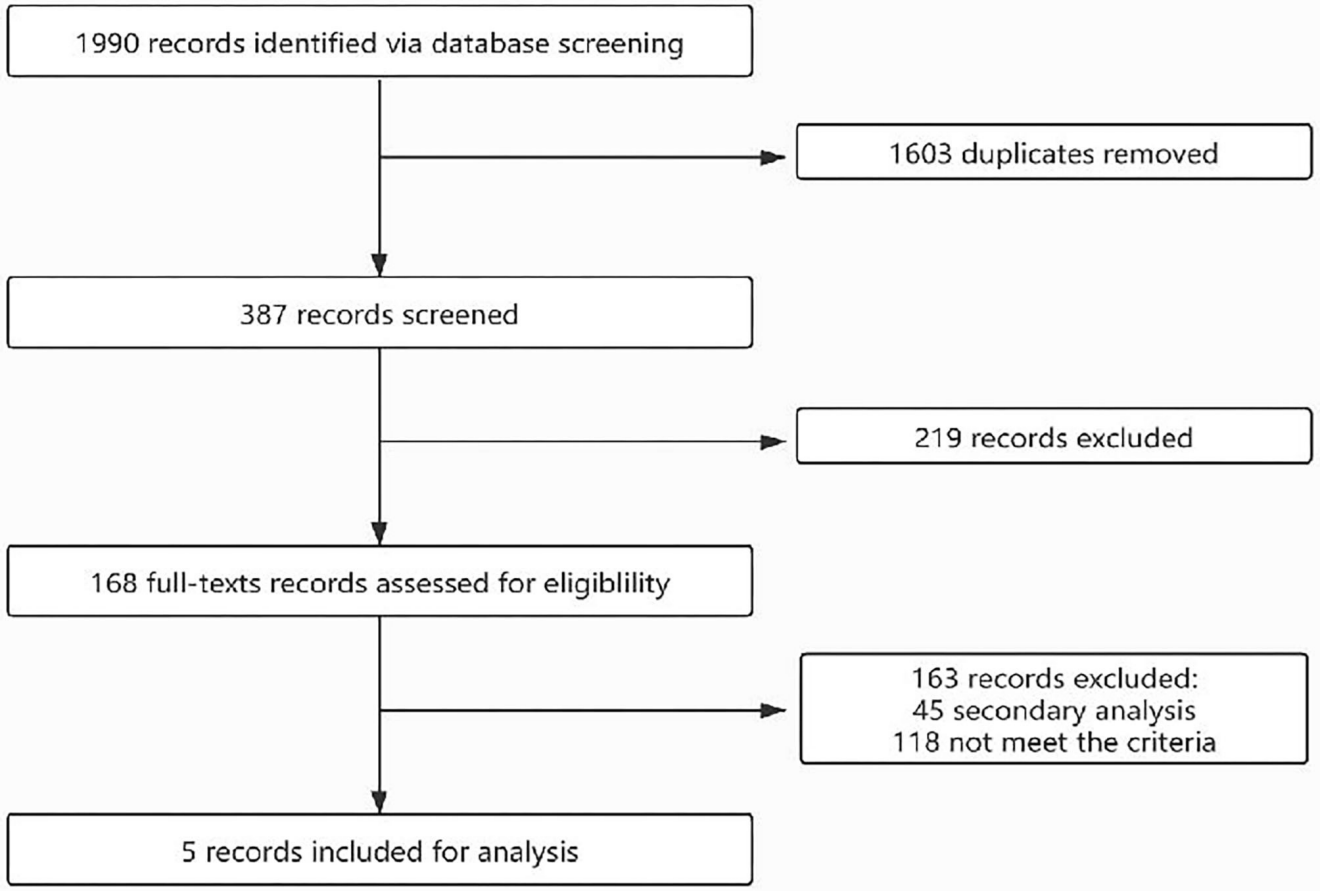
The flow of the selection process.

The flow of the selection process is shown in Figure 1. Initially, 1,990 records were identified through database searching. After duplicate removal and screening *via* title and abstract, 168 records were screened *via* full texts. Finally, five studies were included for analysis and their characteristics are shown in Table 1. A total of 32,827.6 patient-years were observed. The participants were from different countries, and two cohorts were based on a single-center observation. After evaluation with the Newcastle–Ottawa Scales (NOS), the quality of the included studies is good and the bias is acceptable (Table 1).

**Table 1:** Characteristics of the included studies.

**Resources**	**Cohort**	**Country/region**	**Drug**	**Total patient-years**	**Number of women (%)**	**NOS**
Shalom et al. (6)	The Psoriasis Longitudinal Assessment (PSOLAR)	Global	Methotrexate	1,463	52.5	6
Failla et al. (7)	University of Liège hospital medical record database	Belgium	Etanercept, adalimumab, infliximab, rituximab, ustekinumab	4,206	Unknown	6
Dreiher et al. (8)	The database of Clalit Health Services (CHS)	Israel	Alefacept, efalizumab	51	33	7
Kalb et al. (9)	The Psoriasis Longitudinal Assessment (PSOLAR)	Global	Etanercept, adalimumab, infliximab, ustekinumab	17,099	43.2	8
Shalom et al. (10)	The database of Clalit Health Services (CHS)	Israel	Etanercept, adalimumab, infliximab, ustekinumab, methotrexate	10008.6	52.6	8

*NOS, Newcastle–Ottawa Scale*.

**Figure 2:**
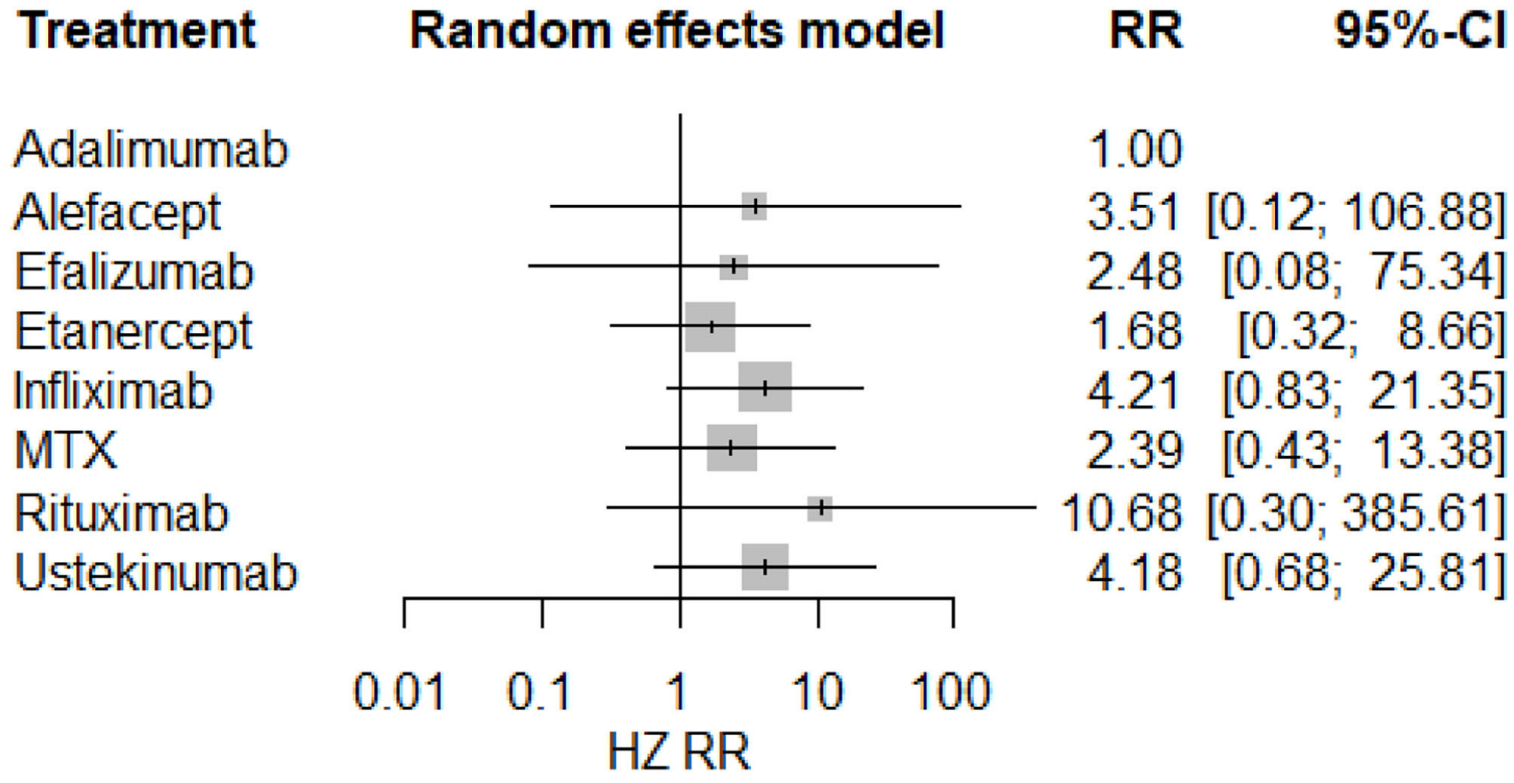
Forest plots of network meta-analysis.

**Figure 3:**
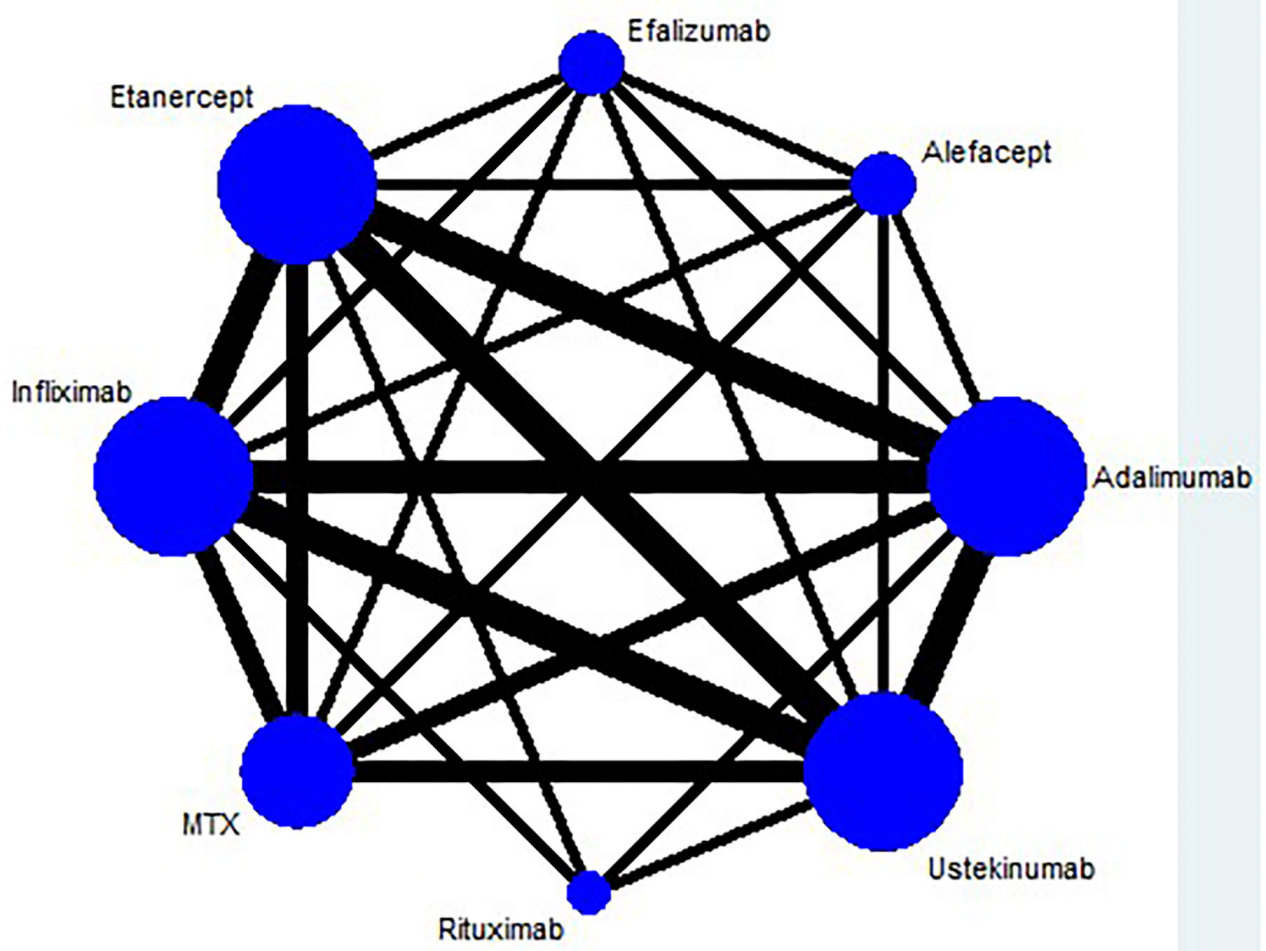
Network plots of network meta-analysis.

**Figure 4:**
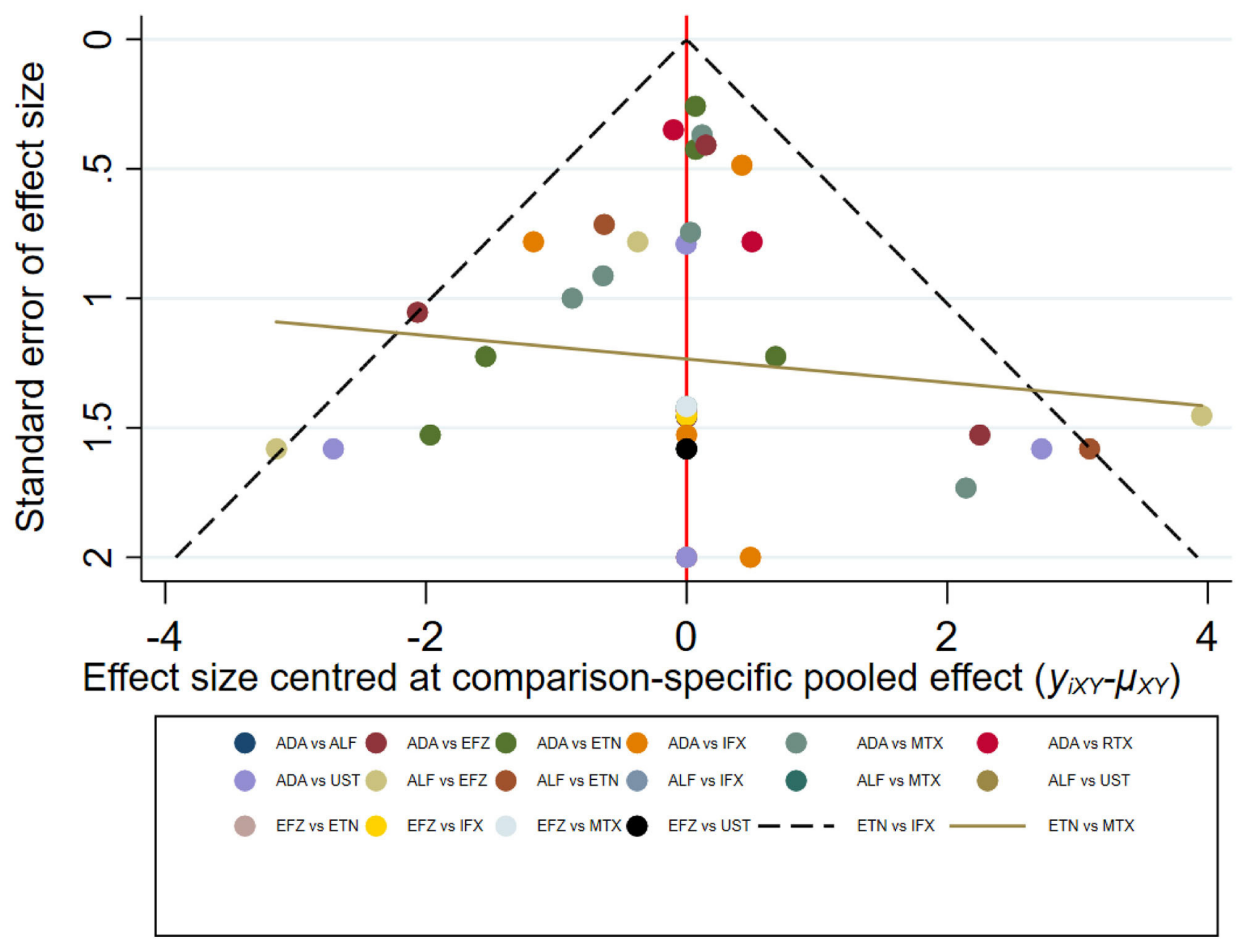
Funnel plot of network meta-analysis.

The result of the meta-analysis is illustrated Figure 2 and network in Figure 3. Since adalimumab accounts for the most patient-years in our analysis (pooled HZ incidence rate = 2.6 per 1,000 patient-years), it serves as the reference during comparison. For biologics targeting tumor necrosis factor-α (TNF-α), patients taking etanercept exhibit a similar risk for HZ compared to those receiving adalimumab (RR = 1.68; 95% CI: 0.32–8.66). Infliximab exhibits the highest risk for HZ (RR = 4.21; 95% CI: 0.83–21.35) among TNF-α inhibitors, although no statistical significance is observed compared to adalimumab. Interestingly, the risk of HZ in patients taking ustekinumab (RR = 4.18; 95% CI: 0.68–25.81), an interleukin-12/23 inhibitor, is similar to infliximab. Some small groups of patients receiving outdated biologics including alefacept and efalizumab were also included in the analysis. No significant difference is observed between those two biologics and adalimumab. Similarly, there are some sporadic patients taking rituximab, a biologic targeting B lymphocyte depletion (3). However, more evidence is needed to confirm the high risk of HZ associated with rituximab (RR = 10.68; 95% CI: 0.30–385.61). Besides, methotrexate was involved in analysis and exhibits a risk similar to infliximab, with no significance (RR = 2.39; 95% CI: 0.43–13.38). The funnel plot suggests that publication bias of included studies is acceptable (Figure 4). The Preferred Reporting Items for Systematic Reviews and Meta-Analyses (PRISMA) statement is included in Supplementary Materials.

## Discussion

In this study, we compared the potential HZ risk among patients receiving monotherapy of seven types of biologics. Nevertheless, our analysis based on several cohorts shows an insignificant difference among the patients receiving adalimumab, alefacept, efalizumab, etanercept, infliximab, rituximab, ustekinumab, and methotrexate.

The application of biologics has brought significant improvement in the management of autoimmune diseases. In the meantime, infections, as a major adverse event, have raised clinicians' attention when deciding to make treatment plans. In 2016, Marra et al. performed a systematic review and meta-analysis 40 eligible RCTs and 19 observational studies to examine the risk of HZ among patients suffering from autoimmune diseases (11). The result from this study indicated that receiving biologics were associated with a higher risk of HZ. However, this study included almost all kinds of autoimmune diseases, leaving out the disease-specific effect. In fact, a large cohort study has suggested that different autoimmune diseases may be associated with different risks of HZ (12). To provide more precise advice for psoriasis management, we must focus on evidences and information that are disease specific.

So far, evidences about HZ risk in psoriatic patients taking biologics are sparse. However, emerging trials have been conducted to evaluate the safety and efficiency of novel biologics among psoriasis patients. Most of them, however, provide clear information about the pathogen type of infections, which may be more indicative for prevention. To evaluate the risk of HZ, we therefore chose real-world studies for analysis. In general, results from cohort studies regarding this issue are contradicting. One study has confirmed the increased risk of HZ among the moderate-to-severe psoriasis patients and proposed its association with the application of immunosuppressive therapies (13). A previous review based on clinical reports, cohort studies, and randomized controlled studies states that the effect of a certain type of biologic remain elusive, except a confirmation of infliximab's contribution to HZ (14). However, a recent systematic review conducted by the Medical Board of the National Psoriasis Foundation proposed that monotherapy of TNF-targeted biologics does not increase the risk of HZ without a quantitative analysis (15). In this study, we pooled the results from a large real-world analysis and further proved that apart from TNF-targeted biologics, interleukin-targeted biologics does not increase HZ risks among psoriasis patients, too. This result will further support the safety of biologics in psoriasis treatment.

Of note, the negative findings of our study do not mean the unnecessity of vaccination. Several limitations must be stated. First, owing to the lack of relevant information, combination therapy of biologics and conventional treatment was not included in this study, which is often associated with more complicated conditions, and the immune dysregulation can be more severe. Also, our results may be restricted by the limited size of the study population. Thus, more efforts must be taken to further determine HZ risk of different therapeutic strategies.

## Conclusion

Based on this analysis, the type of mono-biologic treatment contributes little to the risk of HZ among psoriasis patients. In the future, more real-world evidences are warranted to further investigate HZ risk among psoriasis patients taking biologics, especially combined treatment.

## Data Availability Statement

The raw data supporting the conclusions of this article will be made available by the authors, without undue reservation.

## Author Contributions

ZT: data collection and writing. MS and XC: study design and funding. MS and ZT: data analysis. All authors: data interpretation, revision, and final approval.

## Conflict of Interest

The authors declare that the research was conducted in the absence of any commercial or financial relationships that could be construed as a potential conflict of interest.
